# Epidémiologie et stratégies de riposte contre la COVID-19: l´expérience sénégalaise de 2020 à 2021

**DOI:** 10.11604/pamj.2022.43.204.38290

**Published:** 2022-12-23

**Authors:** Mbouna Ndiaye, Kalidou Djibril Sow, Alioune Badara Ly, Boly Diop, Mady Ba, Adama Faye

**Affiliations:** 1 Programme de Formation en Epidémiologie de Terrain (FETP), Dakar, Sénégal,; 2 Centre des Opérations d´Urgence Sanitaire, Dakar, Sénégal,; 3 Direction de la Prévention, Ministère de la Santé et de l'Action Sociale, Dakar, Sénégal,; 4 Organisation Mondiale de la Santé, Bureau de Dakar, Dakar, Sénégal,; 5 Service de Médecine Préventive et Santé Publique, Université Cheikh Anta Diop de Dakar, Dakar, Sénégal

**Keywords:** Epidémiologie, COVID-19, stratégie, riposte, Sénégal, Epidemiology, COVID-19, strategy, response, Senegal

## Abstract

**Introduction:**

en réponse à la pandémie au SARS-CoV-2 qui a atteint le Sénégal en mars 2020, le pays a mis en place plusieurs stratégies pour contenir sa propagation. Notre objectif était de décrire l'épidémiologie et les stratégies adoptées.

**Méthodes:**

il a été réalisé une étude transversale descriptive des cas confirmés de COVID-19 par RT-PCR du 2 mars 2020 au 30 septembre 2021 au Sénégal. Les données ont été collectées grâce à une revue documentaire et analysées avec les logiciels R et QGIS. Les proportions et les moyennes avec leur écart-type ont été calculées.

**Résultats:**

le Sénégal a enregistré au total 73 782 cas confirmés et 1 859 décès dus au SARS-CoV-2. L'évolution temporelle était marquée par trois vagues épidémiques. L'épidémie était concentrée dans les zones à forte densité comme Dakar (48 656 cas soit 66%), chez les hommes (sex-ratio 1,13) et dans la tranche d'âge 25-34 ans (16 527 cas, soit 22,4%). L´âge moyen des cas était de 43 ans ± 18. L'incidence cumulée nationale était de 428 pour 100 000 habitants et le taux de létalité globale de 2,5% (1 859/73 782). Des stratégies ont été mises en œuvre: formation du personnel, mesures restrictives, gestion des cas à domicile et vaccination. Une proportion de 9,2% (840 154/9 128 453) de la population cible avait reçu 2 doses de vaccin.

**Conclusion:**

l'épidémie était plus répandue dans certains groupes populationnels. Nous recommandons de renforcer les mesures préventives dans les villes à forte densité et de mobiliser les réseaux communautaires pour encourager la vaccination.

## Abstract (en)

**Introduction:**

in response to the SARS-CoV-2 pandemic that reached Senegal in March 2020, the country has put in place several strategies to contain its spread. The purpose of this study was to describe the epidemiology and the strategies adopted.

**Méthods:**

we conducted a descriptive cross-sectional study of confirmed cases of COVID-19 using RT-PCR test in Senegal from March 2, 2020 to September 30, 2021. Data were collected through a literature review and analyzed with R and QGIS software. Proportions and means with standard deviation were calculated.

**Results:**

Senegal has recorded a total of 73,782 confirmed cases and 1,859 deaths from SARS-CoV-2. The temporal evolution was marked by three epidemic waves. The epidemic was concentrated in high-density areas such as Dakar (48,656 cases or 66%), in men (sex-ratio 1:13) and in the age group 25-34 years (16.527 cases or 22.4%). The average age of patients was 43 ± 18 years; the national cumulative incidence was 428 per 100,000 population and the overall case fatality rate was 2.5% (1,859/73,782). Some strategies have been implemented, including staff training, restrictive measures, home-based case management and vaccination. Nine point two percent (840,154/9,128,453) of the target population received 2 doses of vaccine.

**Conclusion:**

the epidemic was spread more widely within some population groups. We recommend strengthening preventive measures in high-density cities and mobilizing community networks to encourage immunization.

## Introduction

Depuis l´identification du premier cas de maladie à coronavirus (coronavirus disease ou COVID-19) dans la ville de Wuhan en Chine, le monde est secoué par une crise sanitaire sans précédent [1]. Face aux répercussions gravissimes sur les sociétés humaines, des stratégies de ripostes diverses parois simples, tantôt complexes ont été développées pour limiter la propagation de la maladie. Il peut s´agir de décision de restriction de liberté, de contrôles frontaliers, de mesures barrières, de vaccination déclaré Comme Urgence De Santé Publique de Portée Internationale (USPPI) par l´Organisation Mondiale de la Santé (OMS) [2,3], le «Severe Acute Respiratory Syndrome Coronavirus 2» (SARS-CoV-2) s´est propagé presque dans tous les pays du monde touchant ainsi plus de 232 millions de personnes et causant environ 4 millions de morts à la date du 30 sept 2021 [3]. De la nature et de l´efficacité de la réponse à l´épidémie, dépendra le taux de létalité qui est très variable, moins de 0,1% à plus de 25% d´après les estimations de l´OMS [4]. Le continent africain comptait plus de 6 millions de cas enregistrés, soit 3% de tous les cas signalés dans le monde et 145 269 décès confirmés de covid à la date du 30 sept 2021 [3]. Le Sénégal a notifié son premier cas confirmé le 02 mars 2020 et depuis cette date, le pays continue d´enregistrer des cas. Devant cette situation épidémiologique inédite, le Sénégal a préparé et mis en œuvre une riposte pour contenir l´épidémie et atténuer les conséquences de la crise sanitaire. Plusieurs fois, des réadaptations se sont révélées nécessaires au gré de l´évolution de l´épidémie. Ainsi des changements ont été opérés dans les stratégies de riposte modulées suivant les différentes vagues. Jusqu'à ce jour, en dehors des rapports officiels de situation, aucune publication n´a encore été faite à notre connaissance sur le déroulement global de l´épidémie au Sénégal durant ces deux années de lutte. Ce faisant, notre travail a pour objectif de décrire l´épidémiologie et la réponse du Sénégal vis à vis du COVID-19 depuis le début.

## Méthodes

**Cadre d´étude**: le Sénégal couvre une superficie de 196 722 km^2^ pour une population de 17 215 428 habitants soit une densité moyenne de 87 habitants au km^2^. Trois régions (Dakar, Thiès et Diourbel) concentrent 47% de la population totale. Le dispositif de surveillance épidémiologique est géré par la Direction de la Prévention avec l´appui du centre des opérations d´urgence. Dans le cadre de la matérialisation de l´approche «une seule santé», les services du Ministère de l´Élevage et des Productions Animales contribuent à la surveillance et au diagnostic des maladies.

**Type et période d´étude**: il s´est agi d´une étude transversale descriptive portant sur les cas confirmés de COVID-19 du 02 mars 2020 au 30 septembre 2021.

**Population d´étude**: l´étude portait sur les cas confirmés de COVID-19 et enregistrés au Sénégal.

**Définition de cas**: un cas confirmé est un patient dont le test de Reverse Transcriptase Polymérase Chain Réaction (RT-PCR-COVID-19) est positif, quels que soient les signes et symptômes cliniques. Le cas confirmé peut être: importé, contact ou communautaire. Le cas confirmé importé: le patient a été diagnostiqué positif au décours d´un voyage entrant au Sénégal. Le cas confirmé «contact»: le patient a été diagnostiqué positif après qu´un lien épidémiologique ait été établi avec un cas positif connu. Le cas confirmé est dit «communautaire» lorsqu´aucun lien épidémiologique n´a pu être fait avec un cas positif.

**Collecte et analyse des données**: nous avons effectué une revue documentaire des données de surveillance du Ministère de la Santé, renseignées à partir des fiches remplies par des prestataires des services de santé. Le contrôle de qualité des données a été effectué à partir des formulaires d´investigation. Les données ont été extraites de la maquette de saisie ensuite nettoyées, corrigées puis analysées avec le logiciel R et QGIS. Nous avons calculé les paramètres ci-après: proportions, indicateurs de morbidité (incidence) et de mortalité (létalité), moyenne et écart-type.

**Considérations éthiques**: l´autorisation de l´utilisation des données était requise auprès des autorités compétentes. La confidentialité a été observée de bout en bout du processus de l´étude.

## Résultats

**Figure 1:**
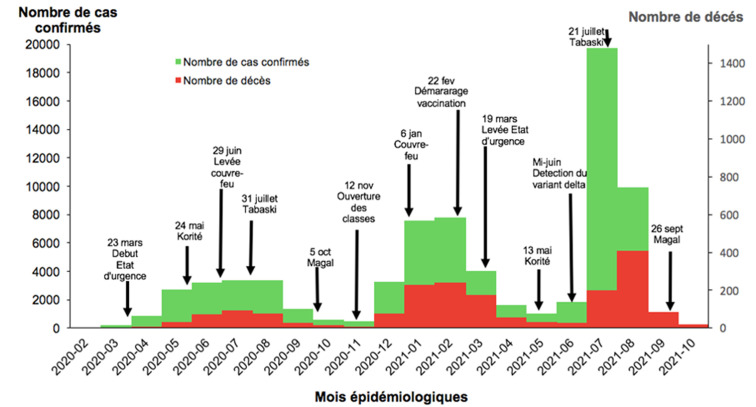
répartition mensuelle des cas confirmés et des décès liés à la COVID-19 au Sénégal du 02 mars 2020 au 30 septembre 2021

**Distribution des cas dans le temps**: au total, le Sénégal a enregistré 73782 cas confirmés par RT-PCR et 1 859 décès liés à la COVID-19 du 02 mars 2020 au 30 septembre 2021. L´évolution temporelle de la pandémie était marquée par 3 vagues. La Figure 1 montre la répartition mensuelle des cas confirmés et des décès liés à la COVID-19 ainsi que les différentes fêtes et évènements survenus au Sénégal durant l´épidémie. La première vague s´étendait de mars 2020 à novembre 2020 (8 mois), avec un pic au mois d´août de 3 371 cas confirmés. Pour les décès, le maximum de 93 était observé au mois de juillet. La deuxième vague débutait au mois de décembre 2020 pour se terminer en mai 2021 (5 mois) avec des pics aux mois de janvier 2021 (7 563 cas confirmés) et de février 2021 (7 805 cas confirmés). Le pic du nombre de décès mensuel était atteint au mois de février 2021 avec 242 morts. La troisième vague commençait au mois de juin 2021 et se poursuivait jusqu´en septembre 2021 (3 mois). Cette vague restait caractérisée par une forte transmission avec un pic de 19 739 cas confirmés au mois de juillet et un maximum de 407 décès enregistrés au mois d´aout.

**Répartition spatiale des cas**: les quatorze (14) régions du pays étaient touchées. En termes d´effectifs, les régions de Dakar (48 656 cas confirmés, soit 66% de l´ensemble des cas), Thiès (7 823 cas, soit 10%) et Diourbel (3 291 cas, soit 4%) étaient les plus affectées par la pandémie. La région de Kaffrine (253 cas, soit 0,3%) était la moins touchée.

### Distribution des cas en terme de personne

**Figure 2:**
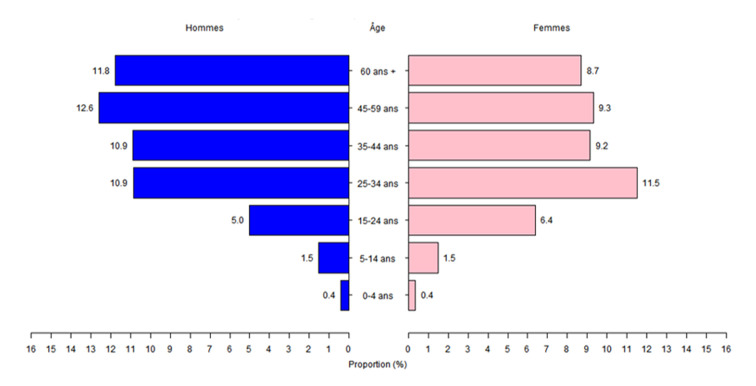
pyramide des âges, par sexe, des cas confirmés de COVID-19 au Sénégal du 02 mars 2020 au 30 septembre 2021

***Selon l´âge et le sexe***: les hommes étaient les plus représentés avec un sex-ratio de 1,13. L´âge moyen des cas était de 43 ans ± 18. Globalement, la tranche d´âge la plus représentée était celle des 25-34 ans avec 22,4% (16 527 cas). Les classes d´âges qui prédominaient étaient celles des 25-34 ans chez les femmes (11,5%) et celles des 45-59 ans chez les hommes (12,6%), (Figure 2).

**Figure 3:**
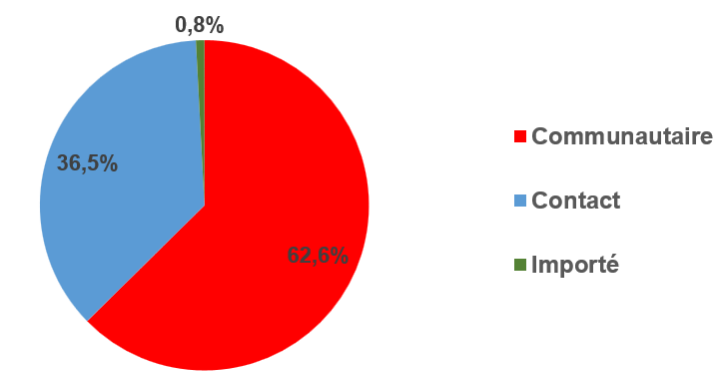
répartition des cas confirmés de COVID-19 au Sénégal du 02 mars 2020 au 30 septembre 2021 selon le type de transmission

***Selon le type de transmission***: la transmission communautaire prédominait largement avec 62,6% des cas (46 217); les cas importés représentaient moins de 1% des cas (608), (Figure 3).

***Selon la symptomatologie clinique***: parmi les cas confirmés, 39% (28 551/73 782) présentaient des signes cliniques. Les principaux signes retrouvés étaient: la toux avec 71% (20 198/28 551), la fièvre 70% (19 953/28 551), le mal de gorge 35% (10 092/28 551) et la rhinorrhée 20% (5 854/28 551).

**Figure 4:**
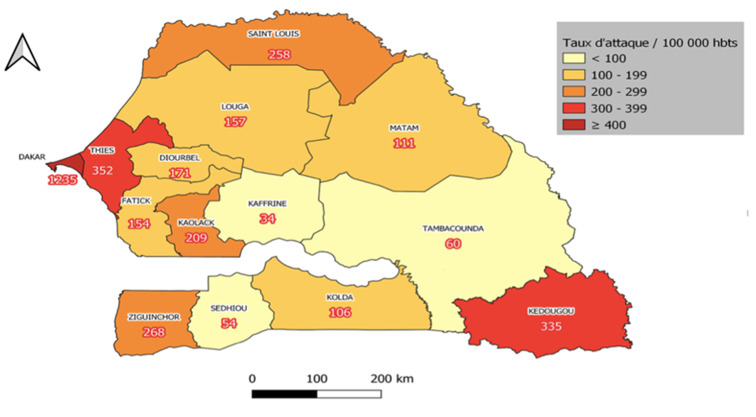
incidence de la COVID-19 par région au Sénégal du 02 mars 2020 au 30 septembre 2021

***Incidence et létalité de la maladie***: l´incidence cumulée nationale de la maladie était de 428 pour 100.000 habitants (73 782/17 215 428) habitants. La région de Dakar avait la plus forte incidence (Figure 4). La létalité globale au niveau national était de 2,5% (1 859/73 782). La proportion de décès était plus importante dans la tranche d´âge 60 ans et plus (1 321/11 683 soit 11,3%), (Tableau 1).

**Tableau 1:** répartition des cas confirmés, des décès et de la létalité liés à la COVID-19 selon les classes d´âges au Sénégal du 02 mars 2020 au 30 septembre 2021

Classe d´âges (ans)	Nombre de cas confirmés	Nombre de décès	Létalité (%)
**0-4**	430	2	0,5
**5-14**	1 715	3	0,2
**15-24**	6 510	23	0,4
**25-34**	12 761	37	0,3
**35-44**	11 427	65	0,6
**45-59**	12 492	259	2,1
**60 et plus**	11 683	**1 321**	**11,3**

### Stratégies de riposte pendant les différentes phases de l´épidémie

**Phase pré-épidémique**: dans le domaine de la coordination, le Comité National de Gestion des Épidémies avait mis en place huit commissions thématiques qui était chargées de définir les stratégies de lutte. Au niveau des frontières maritimes, un contrôle sanitaire systématique était fait pour tous les navires ayant fréquenté une zone affectée. Les mêmes directives étaient appliquées par les régions médicales au niveau des frontières terrestres. Pour les frontières aériennes, un protocole sanitaire visant à réduire les risques de contamination dans les aéroports et les aéronefs exploités au Sénégal était mis en place. Le suivi des voyageurs était assuré par les districts sanitaires en collaboration avec les services de contrôles aux frontières. Sur le plan de la prise en charge d´éventuels cas, quatre hôpitaux était retenus comme structures de référence pour le traitement des malades (Hôpital Principal de Dakar, Hôpital Général Idrissa Pouye, Hôpital Aristide Le Dantec, Service des Maladies Infectieuses et Tropicales de Fann). Les procédures opérationnelles normalisées ont été développées et des séries de formation sur le diagnostic, la prévention et le contrôle des infections étaient organisées. Dans le cadre pharmaceutique, le Ministère de la Santé avait fait des commandes de médicaments et produits essentiels auprès de la Pharmacie Nationale d´Approvisionnement et des fournisseurs locaux et étrangers pour renforcer les stocks déjà existants. Dans le domaine de la communication, experts, journalistes, animateurs et communicateurs traditionnels avaient pris d´assaut les différents media et réseaux sociaux du territoire pour informer les populations sur les précautions à prendre.

### Phase épidémique

***Première vague***: sur le plan politique, les autorités prenaient les premières mesures de lutte contre l´épidémie: fermeture des voies aériennes, maritimes et terrestres, renforcement du contrôle sanitaire aux frontières, interdiction des manifestations publiques, couvre-feu, obligation légale de port de masque, réduction du nombre de passagers dans les transports en commun et arrêt des enseignements scolaires et universitaires, lavage des mains et distanciation physique et sociale. Dans le domaine de la surveillance épidémiologique, la gestion de la COVID-19 a vu la création d´une cellule d´alerte nationale dès le début de l´épidémie avec des numéros d´appels spécifiques. Les Comités de Veille et d´Alerte Communautaire intégrés existant dans certains districts mettaient en œuvre la surveillance communautaire. En matière de prise en charge médicale et psychosociale, la gratuité était assurée aussi bien pour les cas que pour les contacts. Les services sociaux accompagnaient la prise en charge médicale par un soutien psychologique et un appui en kits alimentaires et d´hygiène. Les contacts des cas positifs étaient mis en isolement dans des sites d´hébergement de l´armée, dans de grands hangars publics aménagés à cet effet ou dans des réceptifs hôteliers réquisitionnés. La prise en charge des cas positifs peu ou pas symptomatiques à domicile (PECADOM) devenait une alternative pour faire face à la pression sur le système de soins. Par ailleurs, la prise en charge globale était de plus en plus difficile marquée par: des problèmes d´approvisionnement en matériels médicaux presque entièrement importés de l´étranger et une insuffisance en ressources humaines exacerbée par la contamination des agents de santé. Du point de vue du laboratoire, l´Institut Pasteur de Dakar, centre collaborateur de l´OMS, était le seul laboratoire homologué pour la réalisation des tests Reverse Transcriptase-Polymerase Chain Reaction (RT-PCR-COVID-19) au tout début de l´épidémie avant que l´Institut de Recherche en Santé, de Surveillance Épidémiologique et de Formations ne fasse son entrée dans la liste officielle restreinte. Plus tard, d´autres laboratoires seront intégrés dans le dispositif. Sur le plan de la communication, les leaders communautaires et les stations médiatiques conjuguaient leurs efforts pour sensibiliser les populations sur les mesures de prévention. Malgré les campagnes de sensibilisation, la peur d´être contaminée à la COVID-19 s´était emparée des populations, à tel point qu´elles hésitaient de fréquenter les structures de santé entrainant une baisse dans l´utilisation des programmes prioritaires de santé.

***Deuxième vague***: la résurgence des cas survenait dans un contexte de sollicitation accrue des ressources allouées à la riposte, de démobilisation progressive de la plupart des centres de traitement, de reprise des enseignements et des activités économiques ainsi que dans une atmosphère de relâchement des populations vis-à-vis des mesures barrières. La stratégie de la prise en charge à domicile (PECADOM) prenait de plus en plus de la place et devenait un élément clé de la politique de riposte devant l´afflux massif de malades. Ainsi, la PECADOM était réactualisée et consolidée avec des procédures plus précises. En même temps, la réadaptation à la deuxième vague conduisait à la création de zones tampon dans les districts sanitaires pour remédier à l´engorgement des hôpitaux de référence. La zone tampon consistait en un pavillon d´hospitalisation et de prise en charge temporaire des cas graves en attendant leur évacuation vers les structures dédiées. Pour ce faire, le Ministère de la Santé avait contractualisé avec des prestataires de santé dans l´optique de renforcer les districts en ressources humaines. La vaccination démarrait le 22 février 2021 dans le pays; une démarche progressive et échelonnée était adoptée par le programme chargé de l´immunisation. Elle reposait sur deux aspects principiels: le risque de contamination et la fragilité (comorbidité, âge avancée). C´est pourquoi, elle concernait d´abord les cibles à risques (agents de santé) puis celles fragiles (personnes âgées et/ou porteuses de comorbidité). La vaccination contre la COVID-19 s´adossait sur le système routinier du Programme Elargi de Vaccination (PEV) et s´alignait sur les 3 traditionnelles stratégies (fixe, avancée et mobile) pour atteindre les communautés. Parallèlement à la vaccination les autres moyens de prévention étaient toujours en vigueur même si leur application posait problème. La deuxième vague était aussi marquée par la montée en puissance de la composante laboratoire avec l´homologation et l´enrôlement d´autres laboratoires nationaux qui venaient contribuer aux efforts soutenus de l´Etat dans le domaine du dépistage biologique. Le diagnostic de la maladie était décentralisé par la mise à disposition de laboratoires mobiles et l´utilisation du GeneXpert dans plusieurs régions du Sénégal. Malgré ces avancées, certaines contrées du pays sont encore très éloignées des sites de diagnostic biologique.

***Troisième vague***: durant la troisième vague, la problématique de la transmission des données se posait avec acuité devant le débordement des cas et la surcharge de travail des agents. La transmission des données hebdomadaires se faisait à travers le circuit habituel de la surveillance qui part du niveau opérationnel à celui central. Il existait une multiplicité de plateformes électroniques de transmission des données gérées par différents services du ministère en plus des initiatives régionales propres. Devant l´intensification des cas et particulièrement la recrudescence des décès liée au variant préoccupant «delta» du coronavirus, mis en évidence pendant cette vague pour la première fois, l´option de la densification des structures de prise en charge était prise par les autorités. Au même moment, les autorités avait pris une mesure importante en rendant gratuite la distribution de l´oxygène qui devenait de plus en plus rare, créant des spéculations sur le marché. La diffusion des messages sur les mesures de prévention continuait bien que leur application ne soit pas effective partout. La vaccination était généralisée aux individus de plus de 18 ans. Elle prenait de l´intérêt chez les populations au regard du nombre de décès et des personnes atteintes de plus en plus jeunes. A la date du 30 septembre 2021, 13,7% (1 251 403/9 128 453) de la population cible avaient reçu au moins une dose et 9,2% (840 154/9 128 453) étaient complètement vaccinées (2 doses). Selon la catégorie, la proportion de personnes complètement vaccinées ou taux d´achèvement était de 72% (66 016/91 392) parmi les personnels de santé, de 54% (295 771/548 911) parmi la cible des 19-60 ans et de 60% (190 791/317 834) parmi les personnes âgées de plus de 60 ans.

## Discussion

**Distribution temporo-spatiale**: la maladie progresse au fil du temps sous la forme de flambées qui correspondent aux différentes vagues. De la première vague à la troisième en passant par la deuxième, l´intensité en termes de cas confirmés est allée crescendo, cependant l´amplitude temporelle diminuait. En d´autres termes, la troisième vague a été la plus sévère (pic le plus élevé) mais de plus courte durée alors que la première vague était la moins sévère avec une période plus longue. D´autres auteurs ont rapporté une évolution temporelle similaire dans beaucoup de pays africains et à la date du 26 août 2021 quelques 24 Etats ont connu trois vagues. Tout comme l´Afrique, l´Asie du Sud-Est a subi 3 vagues épidémiques. En revanche, les Amériques, le Pacifique Ouest, la Méditerranée Orientale et l´Europe ont été frappés par au moins 4 vagues [3]. La maladie sévissait essentiellement dans les 3 régions les plus peuplées du Sénégal. Ces trois régions totalisaient les 80% des cas confirmés de l´ensemble du pays. Des recherches antérieures ont montré que quel que soit le pays, ce sont les grands centres urbains qui sont les principaux foyers d´éclosion et d´épicentre de diffusion de la pandémie: en Algérie, ce sont les villes d´Alger, Blida, Oran, Sétif, Bordj-bou-Arréridj, qui sont les plus atteintes par le COVID-19; au Cameroun, Douala et Yaoundé sont les villes les plus affectées; en République du Congo, ce sont Brazzaville et Pointe-Noire; au Gabon c´est Libreville et au Tchad, Ndjamena est la métropole la plus exposée [5,6].

**Distribution des cas en termes de personne**: le sex-ratio était de 1,13 en faveur des hommes. D´autres études ont révélé que les hommes étaient plus touchés. Le sex-ratio trouvé par Nikpouraghdam M. *et al*. [7] en Iran était de 1,93. En Algérie, il variait entre 1,4 et 1,8 selon Ketfi A. *et al*. [8]. En chine, [9] le sex-ratio était de 1,11. La tranche d´âge la plus représentée était celle des 25-34 ans (22,4%). Au Mali, la tranche d´âge de 15-34 ans était la plus représentée avec une proportion de 47,73% de l´effectif des cas confirmés [10]. Selon une étude réalisée au Congo, la tranche d´âge de 30-39 ans était la plus affectée soit 32,12% [11]. En terme de proportion, ce sont les adultes âgés entre 25 et 49 ans qui représentaient la part la plus importante avec 37,9% des cas en Algérie [12]. La prédominance de la maladie dans la tranche d´âge des adolescents et adultes dans la plupart des études peut s´expliquer par le fait qu´il s´agit d´un groupe très mobile et économiquement actif donc exposé à la maladie. Concernant la prépondérance de la transmission communautaire dans notre étude, elle traduisait la détection de cas multiples, ou de groupes de cas dans les communautés, qui ne sont pas directement liés épidémiologiquement aux cas importés ou contacts connus [13]. Elle suppose déjà une enquête épidémiologique rigoureuse et approfondie de la part des équipes de première ligne chargées des investigations. Dans des périodes de forte transmission, les prestataires de santé sont débordés d´où l´intérêt de s´appuyer sur la surveillance à base communautaire avec des relais formés.

La présentation clinique de l´infection au Sars-CoV-2 est très polymorphe avec parfois des formes asymptomatiques (61% dans notre étude). La proportion de formes asymptomatiques est encore débattue, elle se situe autour de 15% selon Byambasuren O. *et al*. [14]. Parmi 634 cas confirmés d´infection à SARS-CoV-2, 17,9% étaient asymptomatiques dans la série des 3711 passagers ou membre de l´équipage du bateau de croisière «Diamond Princess», restés en quarantaine au port de Yokohama (Japon). A la différence de notre étude, il s´agit d´un modèle quasi expérimental de l´infection par le SARS-CoV-2 où les évènements ont lieu dans un site unique [15]. Dans une autre étude de 24 patients infectés lors de contacts intra-familiaux et hospitalisés pour surveillance et monitorage des symptômes, 29,2% de patients ne présentait ni symptômes, ni anomalies scannographiques [16]. Le COVID-19, étant une virose à tropisme essentiellement pulmonaire, les symptômes sont dans la majorité des cas de type respiratoires dans notre étude. D´autres recherches ont abondé dans le même sens concernant la symptomatologie. Selon Pascarella G. *et al*. les symptômes le plus fréquemment rapportés étaient la toux (75%), plutôt sèche, la fièvre (50%) et la dyspnée (30%) [17]. Les signes cliniques retrouvés étaient le plus fréquemment de la fièvre (166 patients, 76%), de la toux (146 patients, 66%) et une dyspnée (137 patients, 62%) dans la série de Sixt T. *et al*. [18].

**Incidence et létalité de la maladie**: le Sénégal, à l´image des pays africains affichait une incidence relativement faible (428/100 000). Il faut rappeler que les malades comptabilisés pendant notre période d´étude représentaient les cas confirmés dans les structures sanitaires et rapportés au ministère de la Santé. L´incidence était de 234 en Côte d´ivoire, 211 en Guinée et 304 pour 100 000 habitants en Ethiopie [3]. Pour ce qui est de la létalité, elle était de 2,5% au Sénégal, 1,02% en Côte d´ivoire, 1,24% en Guinée, et 1,6% en Ethiopie [3]. Comparé aux pays développés, le continent africain est relativement peu touché [19,20]. Pourtant des scenarios catastrophiques avaient prédit le pire dans le continent, fondés essentiellement sur la faiblesse des systèmes de santé, l´insuffisance de la protection sociale, la charge de morbidité lourde des endémies préexistantes (VIH/SIDA, paludisme, tuberculose), le manque de ressources budgétaires et la gouvernance inappropriée. Bien que ce présage soit effarant, le Sénégal tout comme les Etats africains, a su résister aux déferlantes vagues de coronavirus du moins jusqu'à ce jour. Différentes théories ont été avancées pour tenter d´expliquer le phénomène. La population africaine est jeune avec un âge médian de moins de 20 ans, et il semble que les jeunes présentent des formes asymptomatiques ou peu symptomatiques beaucoup plus souvent que les personnes âgées, qui ont un risque significativement plus élevé de contracter des symptômes graves et de décéder [21,22]. Ceci est d´ailleurs confirmé dans notre étude puisque la létalité était plus importante dans la tranche d´âge 60 ans et plus.

**Stratégies de riposte**: comme au Sénégal, d´autres études ont rapporté la mise en œuvre de mesures de restriction des libertés pour combattre l´épidémie dans plusieurs pays du monde [5,6,13]. Au fil du temps, le couvre-feu était abandonné par les autorités gouvernementales pour atténuer les effets délétères du COVID-19 sur l´économie du pays et surtout abaisser la tension sociale sous-jacente. Les réadaptations des mesures selon le cours de l´épidémie et la situation socio-économique étaient observées dans beaucoup de pays durant l´évolution de la pandémie [13,19]. Comme dans notre travail, d´autres auteurs ont rapporté une baisse de la fréquentation des structures de santé par les populations [23,24]. La diminution de la couverture en soins fait redouter une résurgence d´autres maladies. Pourtant, les stratégies mises en œuvre jusque-là au niveau des pays en développement pour atteindre les objectifs du millénaire pour le développement liés à la santé avaient permis de faire des progrès remarquables dans la réduction de la charge de morbidité et de mortalité des maladies endémiques. Pour ce qui est de la multiplicité des plateformes de gestion des données, elle constituait une surcharge de travail et une perte de temps énorme pour les agents de santé, en effet les mêmes informations étaient demandées par les différentes directions du ministère. La proportion de personne complètement vaccinée contre la COVID-19 était en deçà des objectifs fixés. La faiblesse de la couverture vaccinale doit être analysée sur deux angles: la disponibilité du vaccin et son acceptabilité. Si dans les pays développés, le problème se pose essentiellement du point de vue de l´acceptabilité, dans la plupart des pays pauvres, la difficulté est liée d´abord à un manque de vaccin. Néanmoins, la problématique de son acceptabilité constitue une préoccupation majeure des systèmes de santé souvent liée à la peur des effets secondaires [25]. Le rôle de la communauté a été déterminant dans la lutte contre la maladie par la participation de leaders locaux coutumiers et religieux, de marraines de quartiers et de relais. Le même dispositif pourrait être mis à contribution pour combattre les rumeurs infondées sur la vaccination, propagées à travers les réseaux sociaux et améliorer ainsi la couverture vaccinale encore loin de l´objectif national de 70%.

## Conclusion

L´épidémie de COVID-19 a évolué sous la forme de 3 vagues entrecoupées de périodes d´accalmie. La maladie touchait préférentiellement: les régions densément peuplées, la tranche d´âge 25-34 ans et le sexe masculin. Nous recommandons entre autres de: i) intensifier les mesures de prévention particulièrement dans les villes à forte densité populationnelle; ii) passer à l´échelle la mise en place des Comités de Veille et d´Alerte Communautaire intégrés dans les districts pour l´investigation en particulier des cas communautaires et le suivi des contacts; iii) mettre en place une plateforme unique de gestion des données et améliorer leur complétude; iv) assurer une dotation suffisante en matériel médical et ressources humaines au regard des déficits constatés dès la première vague; v) mobiliser les réseaux communautaires pour une adhésion des populations à la vaccination; vi) évaluer l´impact du COVID-19 sur les programmes prioritaires de santé publique.

### Etat des connaissances sur le sujet

- Déclaré comme urgence de santé publique de portée internationale, le SARS-CoV-2 s´est propagé presque dans tous les pays du monde;
- Des stratégies de ripostes diverses parois simples, tantôt complexes ont été développées pour limiter la propagation de la maladie;
- Plusieurs fois, des réadaptations de stratégies se sont révélées nécessaires au gré de l´évolution de l´épidémie.

### Contribution de notre étude à la connaissance

- L´étude a présenté la dynamique de l´épidémie de COVID-19 au Sénégal au cours d´une période de 2 ans;
- L'épidémie était plus répandue dans certains groupes populationnels bien déterminés;
- L´étude décrit l´expérience du Sénégal à travers les différentes stratégies mises en œuvre dans le pays; nous avons recommandé de renforcer les mesures préventives dans les villes à forte densité et de mobiliser les réseaux communautaires pour encourager la vaccination.

## Remerciements

Nous tenons à exprimer notre gratitude au personnel du Centre des Opérations d'Urgence Sanitaire (COUS) pour leur appui technique à la réalisation de cette étude.
